# Macrodiversity Reception with Distributed Hard-Decision Receivers for Maritime Wireless Sensor Networks

**DOI:** 10.3390/s20143925

**Published:** 2020-07-15

**Authors:** Weigang Chen, Dongming Sun, Changcai Han, Jinsheng Yang, Feng Gong, Wei Wang

**Affiliations:** 1 School of Microelectronics, Tianjin University, Tianjin 300072, China; sundm@tju.edu.cn (D.S.); cchan@tju.edu.cn (C.H.); jsyang@tju.edu.cn (J.Y.); 2 Joint Laboratory for Ocean Observation and Detection, Pilot National Laboratory for Marine Science and Technology, Qingdao 266237, China; fgong@qnlm.ac (F.G.); wwang@qnlm.ac (W.W.)

**Keywords:** wireless sensor networks, macrodiversity, equal gain combining, hard-decision

## Abstract

Maritime wireless sensor networks are considered to be the primary means of monitoring methods in the marine environment. The transmission between sensor node and sink node in maritime wireless sensor networks is usually unreliable due to the harsh propagation environment. To extend the transmission range or to enhance the transmission reliability between sensor nodes and sink node, we propose a macrodiversity reception scheme in the sink node equipped with distributed multiple hard-decision receivers. Multiple receivers are divided into several clusters and placed at different locations to receive different signal copies suffering from different fadings. Furthermore, a cascaded combining strategy based on hard-decision information is used to reduce the overall complexity of receiving side. The experimental results in the ocean scenarios show that the macrodiversity reception scheme with two antenna clusters has a transmission gain of 3–4 dB compared with the single antenna reception when the package loss rate is $10^{-2}$. The study casts a new method for reliable transmission in maritime wireless sensor networks using commercial transceivers which can only output hard-decision results.

## 1. Introduction

Maritime wireless sensor networks generally consist of considerable sensor nodes floating on the sea, which are randomly deployed in a particular region to acquire various types of environmental parameters and transmit information to the sink node for monitoring marine environment [1,2]. Compared with traditional marine monitoring methods, maritime wireless sensor networks have the advantages of low cost, easy deployment and good real-time observation [3,4,5,6]. They have attracted great interest of researchers and been widely applied in marine mammals monitoring [7], water quality monitoring [8,9,10,11] and marine engineering [12] in recent years. However, a lot of error packages in the sink node can affect network performance seriously due to the harsh channel environment and limited resource of the sensor node.

Recent advancements in technology focus on further reducing package error rate (PER) from sensor node to sink node, such as channel coding [13], retransmission mechanisms [14,15], and multipath routing protocols [16,17,18]. The channel coding can recover random errors by inserting some redundancy check bits in the transmission sequence. The retransmission mechanisms improve the reliability of transmission by feeding back the status of packages to the previous node. However, in the harsh ocean scenarios, a lot of retransmissions not only accelerate energy consumption of node, but also cause network congestion. Multipath routing protocols use the network topology effectively and transmit package through multiple paths to reduce PER. However, the package is transmitted through multiple paths, which may accelerate energy consumption of intermediate nodes. Improving transmission reliability without extra energy consumption is a main problem in maritime wireless sensor networks. To cope with this issue, a possible solution is to incorporate the idea of diversity combination in the sink node. Diversity is considered to be a simple method to improve transmission reliability, which employs multiple distributed antennas against fading without complex signal processing in wireless communication. Generally, if multiple antennas are far apart and installed on different receivers, it is called macrodiversity. In the macrodiversity system, the receiver does not just reduce the effects of fast fading effectively, but also combat shadow fading additionally. In maritime wireless sensor networks, macrodiversity in the enhanced receivers has outstanding advantages in improving transmission performance, which does not need the sensor node to adopt complex processing methods and is quite suitable for sensor node sending to data to the sink node.

A macrodiversity reception strategy is used in various applications to improve transmission reliability in recent years. In cellular systems, co-channel interference (CCI) and blocking objects (blockages) between the transmitter and receiver can seriously affect the quality of wireless communication [19,20]. The symbol error rate performance of macrodiversity reception scheme based on maximal ratio combining (MRC) in flat rayleigh fading with CCI was analysed in [21,22]. For the random blockages in cellular system, macrodiversity is considered to be an inexpensive and simple solution. Multiple base stations are placed at different locations, which can increase the probability of line-of-sight (LOS) links between the transmitter and receiver. To guarantee the probability of LOS, a framework was proposed to determine the number of base stations in [23]. In live broadcasts system of road races, a minimum-mean-square-error macrodiversity reception system using distributed remote antennas and radio-on-fiber links was used for improving transmission reliability. Considering the propagation delay differences among diversity branches, a practical delay difference correction technique was proposed to keep signal continuity in [24]. It is well known that the transmission performance is better as the number of antennas increases, but the complexity of the signal processing on the receiver becomes higher. A macrodiveristy system composed of a macrodiversity receiver and two microdiversity receivers was proposed in [25], since it had lower implementation complexity than other macrodiversity systems. The macrodiversity reception scheme using microdiversity receiver as distributed remote antenna was considered to be effective against both large-scale fading, such as shadowing and path loss, and small-scale fading, such as rayleigh fading. Many valuable results have been reported on the level crossing rate (LCR) [26,27] and signal moments [28,29] at the output of the macrodiversity reception system. Recently, the implementation of the macrodiversity schemes was mature when it was used in the traditional wireless networks. However, its applicability in practical wireless sensor networks to achieve full benefits is still in its infancy [30]. The main issue is the limited computing resources in the sink node. Therefore, it is important to further reduce the implementation complexity of macrodiversity system to adapt to the hardware constraints of the sink node.

In this paper, a low complexity macrodiversity reception scheme using multiple receivers which can only output hard decision result is proposed for maritime wireless sensor networks. The scheme is based on a macrodiversity reception architecture consisting of several local convergence modules and a central processing module, which can reduce the effects of fading effectively. Each local convergence module equipped with four radio frequency (RF) antennas is placed in a remote place to receive data. The central processing module aggregates the output of each local convergence module to the processor, and completes the combining of multiple signals on the processor. To reduce the computing complexity of processor in convergence center, a cascaded combining scheme is provided to distribute the computing load of the sink node to each local convergence module. Furthermore, we build a maritime wireless sensor networks macrodiversity reception platform and conduct experiments to analyze the performance of proposed macrodiversity reception scheme. The experimental results show that the macrodiversity reception with two clusters using four antennas scheme has a transmission gain of 3–4 dB compared with the single antenna reception scheme when the package loss rate is $10^{-2}$ and has greater gain than single cluster using eight antennas reception scheme.

The remainder of this paper is organized as follows. Section 2 gives a brief overview of the proposed macrodiversity reception scheme. In Section 3, we describe the reliable transmission method of macrodiversity reception in detail. In Section 4, we conduct experiments in different scenarios and analyze the experimental results in terms of PER and frame error rate (FER). Conclusion is given in Section 5.

## 2. Proposed Macrodiversity Reception Architecture for Maritime Wireless Sensor Networks

**Figure 1:**
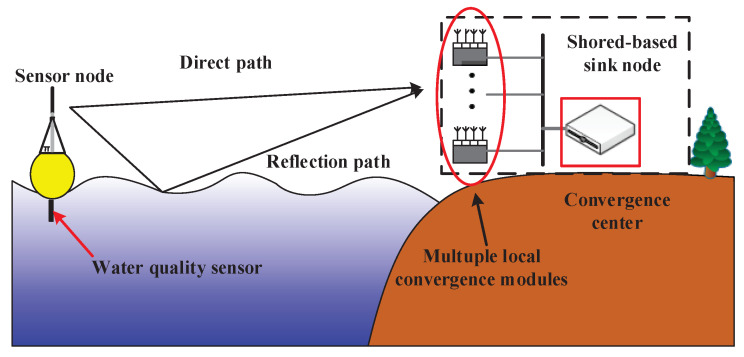
Proposed maritime wireless sensor networks architecture.

The maritime wireless sensor networks macrodiversity reception platform designed in this paper is mainly composed of sensor node and shore-based sink node. The architecture of maritime wireless sensor networks is shown in Figure 1. The sensor node floating on the sea collects the seawater information in real time and transmits it to the shore-based sink node in a fixed time interval. The shore-based sink node is based on macrodiversity reception scheme and consists of multiple local convergence modules and convergence center. A plurality of local convergence modules are placed at different locations on the shore to receive package. The convergence center connects with multiple distributed reception nodes and receives packages from each distributed reception node for combining to against multipath fading effectively.

**Figure 2:**
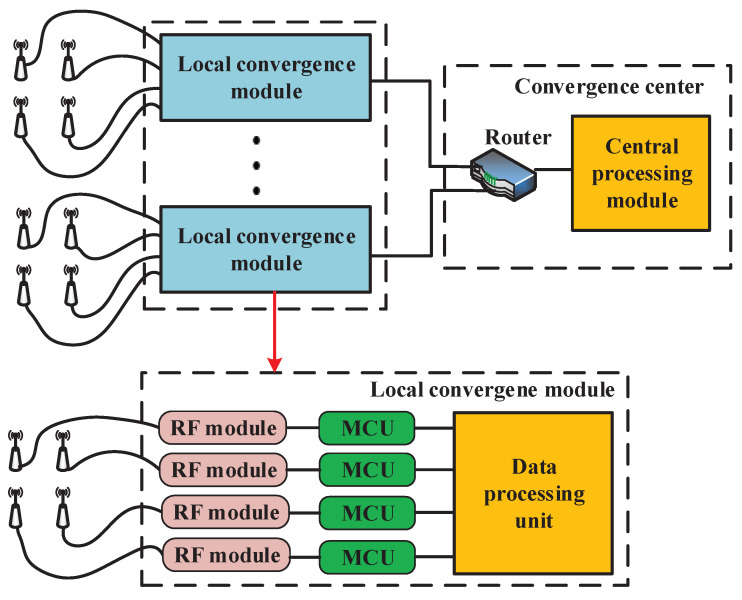
Architecture of the proposed macrodiversity reception system.

The proposed macrodiversity reception scheme indicates that the sink node is mainly composed of mutiple local convergence modules and a convergence center. Each local convergence module equiped with four antennas is based on the diversity reception scheme to against fast fading. The convergence center connects with multiple local convergence modules via fiber or ethernet and receives packages from every local convergence module. Each local convergence module should be placed at a different location to realize macrodiversity reception scheme. The architecture of the proposed macrodiversity reception system is shown in Figure 2. The local convergence module consists of four RF modules, four lower-power microcontroller units (MCU) and a data processing unit. The RF module configured by MCU receives packages from sensor node. The MCU connects with RF module via serial peripheral interface (SPI) and reads packages received by RF module. The data processing unit should be a low-power microprocessor and connects with MCU through serial port. It buffers and processes the packages received by RF module and sends the data combined to convergence center via optical fiber or ethernet. In the convergence center, a router or switchboard is necessary, which connects with multiple local convergence modules and achieves transmission from multiple local convergence modules to the convergence center. The data processing module connnects with the router or switchboard via ethernet to receive and processes packages from different local convergence modules.

## 3. Implementation Method of Proposed Macrodiversity System for Maritime Wireless Sensor Networks

In this section, we introduce the reliable transmission strategies of the macrodiversity reception scheme in detail. First, the frame format and reliable transmission scheme in the sensor node are designed. Second, the detailed steps of identifying the coded frame ID and transmission package ID using majority-voting decision in the receiver are introduced. Finally, a cascaded combining scheme is proposed, and its specific implementation process is explained in detail.

### 3.1. Transmission Frame and Package Formats for Macrodiversity Reception

To support the macrodiversity reception of data packages from sensor nodes, special frame or package formats are devised. In this part, we detail the formats and explain our design motivations.

First, the information frame for proposed macrodiversity reception scheme comprises of three segments, i.e., the node ID, the user information and cyclic redundancy check (CRC) value. The length of whole information frame is 20 bytes. The information frame design mainly considers the user requirements and the parameters of the turbo codes in our design. Therefore, we choose the information code length as 20 bytes. Node ID is very important for many applications and two bytes are allocated for this part. The size of user information is 16 bytes. In our specific experiments, we use water quality sensor information and global positioning system (GPS) information as user information. The water quality sensor information occupies 6 bytes, while the GPS information occupies 10 bytes. The CRC value is computed based on the first 18 bytes in this information frame.

Second, in order to explore the time diversity and to cancel the random noise, the turbo code and the channel interleaver are used for each information frame [31,32]. The code length of the turbo code is 42 bytes with the information part 20 bytes. After encoding, the whole turbo codeword is interleaved in bit level in order to adapt to the transmission schemes with four small packages.

**Figure 3:**
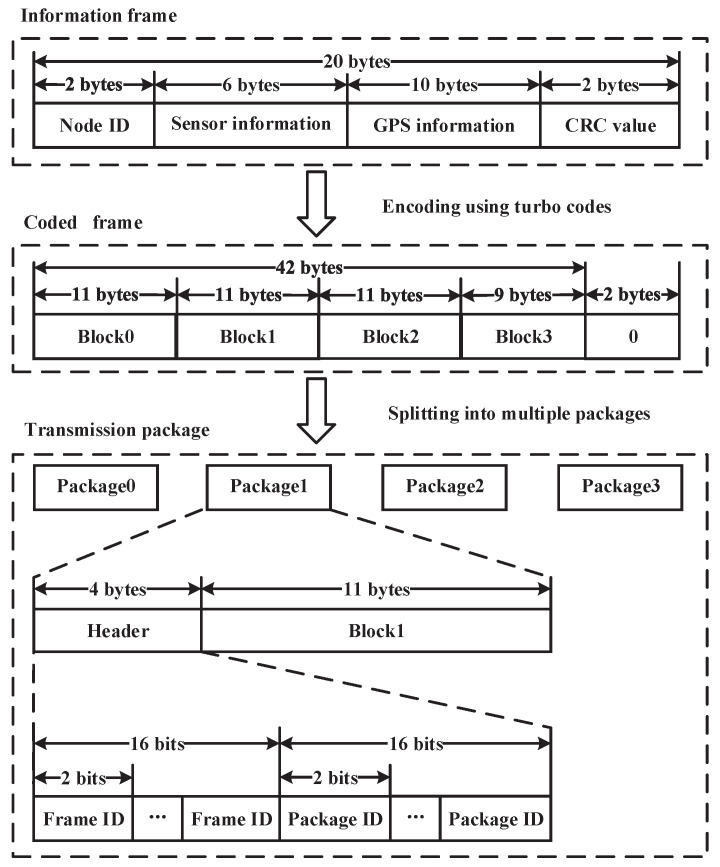
The frame or package formats for macrodiversity reception.

Third, considering the wireless transmission conditions and the chosen transceiver (CC1101), we decompose the turbo codeword into four parts. In this design, on the one hand, the time diversity is obtained by sending the whole codeword with four independent packages. On the other hand, a moderate package length is appropriate for wireless sensor network using the transceiver CC1101 [33,34,35]. The specific decomposition method is shown in Figure 3. It can be observed that two bytes are padded using zeros.

Fourth, the final four transmission packages are formatted into a single information frame. Each coded frame is divided into four independent transmission packages. To identify the packages from one coded frame, the transmission package ID and the coded frame ID are embedded in a transmission package. To guarantee the reliability of the coded frame ID and transmission package ID, repetition coding is used. That is, the two IDs are repeated. Specifically, the coded frame ID and the transmission package ID are repeated eight times, respectively. In our design, the coded frame ID is only used to distinguish packages of the same package ID in different frames, and two bits is used to label four consecutive frames. The package ID is also two bits. Therefore, an eight-fold repetition coding is enough to guarantee the reliability. The specific format is illustrated in Figure 3.

### 3.2. Identifying Coded Frame ID and Transmission Package ID

Considering that the sensor data should be recovered from the coded frame, the coded frame ID and transmission package ID both need to be identified and aligned from different receivers.

The identification scheme of the coded frame ID and transmission package ID is implemented using majority-voting decision algorithm, which classifies all candidate IDs and determines the ID with the most candidate IDs as the current ID. When local convergence module receives the transmission packages, processor needs to extract the header of the received package and identify the coded frame ID and transmission package ID. The specific steps of identifying coded frame ID are as follows:

(1) Extract the information sequence of the first 16 bits in the information header to obtain the coded frame ID sequence;
(2) Generate a coded frame ID every two bits using the unmapping rule {00→0, 01→1, 10→2, 11→3} and count the occurrences of each coded frame ID;
(3) Set the coded frame ID with the most occurrences to the current coded frame ID.

The sequence containing transmission package ID is located in the last 16 bits of the information header, and the identification process is the same as that of the coded frame ID.

### 3.3. Cascaded Combining Scheme

Diversity combining is the core of diversity technology, which determines the combining gain and complexity of diversity system. The traditional diversity combining scheme mainly includes selection combining (SC) [36], equal gain combining (EGC) [37] and MRC [38]. The MRC needs to estimate channel state information (CSI) of each diversity branch strictly and calculate weights based on current CSI. The SC only selects one diversity branch with the largest signal-to-noise ratio for the output of combining. When the number of antennas is large, the combining gain of SC is low. The EGC adds the signals of all the diversity branches with the same coefficient without the need to estimate the CSI of each diversity branch, which can be implemented based on hard decision information and is suitable for wireless sensor networks with lower computational complexity.

**Figure 4:**
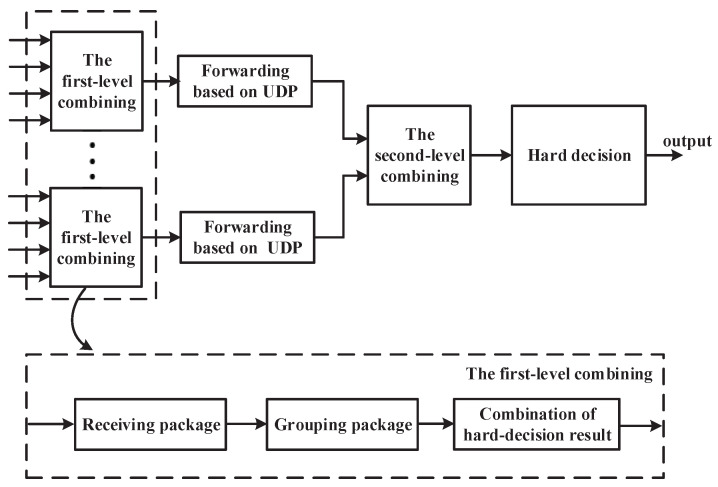
Proposed cascaded combining scheme.

The proposed macrodiversity reception scheme provides a cascaded combing scheme to distribute the computing complexity of the convergence center to each local convergence module, which can effectively reduce the demand for computing resources of sink node. The scheme consists of two diversity combinings. The first-level diversity combining is completed on the local convergence module, and its output is sent to the convergence center based on the user data protocol (UDP) for the second-level diversity combining. The specific process of the cascaded combining scheme is shown in Figure 4.

The receiving and combining of multi-channel packages are finished in the first-level diversity combining. To guarantee the robustness of combining, we need to set two buffers size of M×N, where *M*, *N* represent the number of antennas in the local convergence module and packages in a coded frame respectively. The first buffer is used to cache the packages received by the RF module, and the second buffer is used to cache the packages with the same coded frame ID for grouping and combining. The specific process of the first-level diversity combining includes receiving stage of packages, grouping stage of packages, and combination stage of hard-decision results. Among them, the receiving stage of packages completes receiving and buffering of packages. The grouping stage of packages achieves grouping and transforming of packages. Finally, in the combining stage of hard-decision results, the processor completes EGC scheme of packages and assembles combined packages into a frame. The specific process of each stage is as follows.

(1) Receiving packages. In this stage, the processor receives the hard decision results output by the four RF modules and stores them in the first buffer cyclically. When the first buffer is saturated, the processor identifies coded frame ID of each package in the first buffer and determines the current coded frame ID using majority-voting decision algorithm, firstly. Then, the processor stores the package with the current coded frame ID into the second buffer for combining. Finally, these packages with current coded frame ID in the first buffer are cleared.
(2) Grouping packages. In this stage, the processor divides the buffered packages into four groups according to the transmission package ID in the second buffer and stores the packages into the corresponding group. The packages within each group are then converted into binary sequences.
(3) Combination of hard-decision results. In this stage, processor adopts the EGC strategy to complete the combination of the information sequences in each group. First, the processor maps the information sequence in the group into a bipolar sequence using the mapping rule {0→−1, 1→1}. Second, the corresponding positions of the mapped sequences in the group are summed to generate a combined information sequence. Thirdly, the information sequence generated by each group is reassembled into an information frame in the order of the transmission package ID. Finally, the current coded frame ID in receiving stage of packages is inserted into the header of new information frame to facilitate the identification of the second-level diversity combining.

The second-level diversity combining is implemented on the convergence center, which completes the convergence and combining of multiple frames from different local convergence modules. The second-level diversity combining includes convergence stage and combining stage. In convergence stage, the processor is first to set a fixed internet protocol (IP) address and port to inform the local convergence node of the destination address firstly. Then, the processor creates and binds a local network socket to receive and buffer information frame. When the processor receives the information frame, a coded frame ID detection is necessary. The coded frame ID detection is mainly used to determine whether the current information frame transmission is completed by compared to the previous coded frame ID. When the coded frame ID changes, the package with different coded frame ID is preserved and packages in the cache are combined by EGC strategy. In combining stage, the symbols of corresponding positions in the received information frames are added and a weighted sequence is obtained firstly. Then, the weighted sequence is hard-judged to obtain the hard decision binary information sequence. Finally, the information sequence is put into the turbo decoder to recover the original frame and a CRC check is performed to determine the accuracy of the package.

## 4. Experiment Setup and Performance Analysis

In this section, we construct a maritime intelligent observation platform and conduct experiments to validate the performance of macrodiversity reception scheme in different scenarios. First, the experiment platform is introduced. Second, we analyze the performance of macrodiversity reception scheme using different number of antennas and the performance of diversity scheme using different antenna placement strategies separately in the park scenarios. Finally, experiments are carried out in the ocean scenarios to verify the transmission gain of the proposed macrodiversity reception scheme.

### 4.1. Experiment Platform

The macrodiversity reception scheme is evaluated by four experiments. In our experiments, we use a maritime intelligent observation platform as our testbed. The maritime intelligent observation platform is a communication buoy, which is designed by our researched group. The maritime intelligent observation platform includes hardware and software system of sensor node and buoy structure.

**Figure 5:**
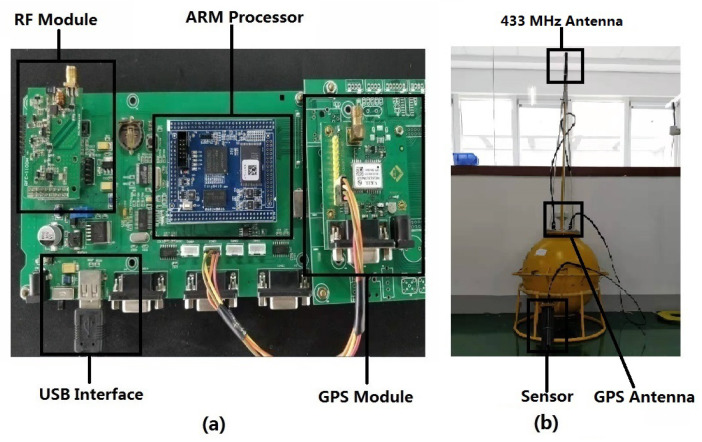
The sensor node in buoy. (**a**) The hardware structure of sensor node. (**b**) The maritime communication buoy.

The hardware of sensor node is mainly composed of an information collection unit, an ARM processor and an information transmission unit. Figure 5a shows the hardware structure of sensor node. The information collection unit is composed of a GPS module and a multi-parameter water quality sensor, collecting the location information of sensor node and water quality parameters. The information transmission unit is composed of a MCU and a RF module. The MCU configures the RF module to send and receive package through the SPI port. The workflow of sensor node mainly consists of three steps. First, the ARM processor sends the request frame to the information collection unit and checks the received package by the CRC value. Second, the ARM processor assembles the received sensor parameters into a frame and performs channel encoding and interleaving. Finally, the coded frame is divided into four packages, which are sent to the information transmission unit via serial port and transmitted in a fixed time interval by the RF module sequentially.

The maritime communication buoy designed in this paper is composed of a 433 MHz RF antenna, an antenna feeder tube, a fixed flange, a GPS antenna, an equipment bay and a counterweight ring. The actual picture is shown in Figure 5b. The 433 MHz antenna feeder is connected to the RF module in the equipment bay through the antenna feeder tube. The bottom of the antenna is designed as a structure of screw nut, which can be directly fixed to the feeder tube through threads during installation. We add a rubber pad between the antenna and the feeder tube to guarantee the watertightness of the buoy. The feeder tube is fixed on the cover of equipment bay by flange, and the position where the feeder tube is connected to the cover of the equipment bay need to be sealed. The GPS antenna interface is also designed on the cover of the equipment bay, and it needs to be sealed during installation. The bottom of the buoy is equipped with a counterweight ring, which can provide optical the drifting monitoring with a light load or the fixed-point monitoring with the heavy load.

**Figure 6:**
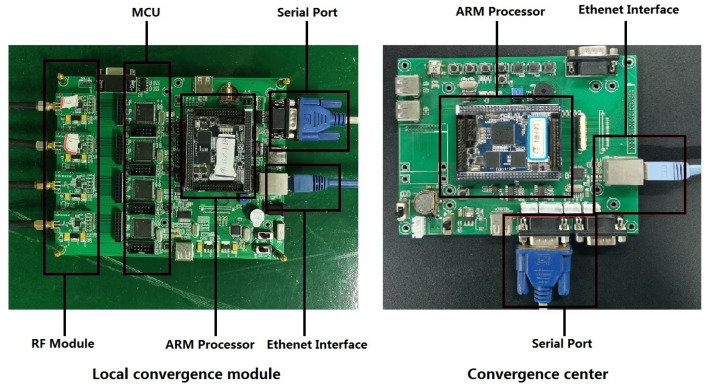
Local convergence module and convergnence center.

The structure of the sink node has already been explained in the Section 2. Based on proposed macrodiversity reception structure, we design the printed circuit board (PCB) of local convergence module and convergence center. In the local convergence module, the RF module and the ARM processor are detachable to facilitate the maintenance and update of the equipment. The local convergence module connects with the convergence center via ethernet. The local convergence module and convergence center are shown in Figure 6.

The RF module of sensor node and sink node is based on wireless RF module CC1101 (Texas Instruments) for transmission, which is configured to send and receive package by the C8051F120 MCU. The parameters of CC1101 is shown in Table 1. The data processing unit of sensor node and sink node is based on tiny6410 microprocessor for signal processing uniformly.

### 4.2. Experiment Results and Analysis in the Park Scenarios

**Figure 7:**
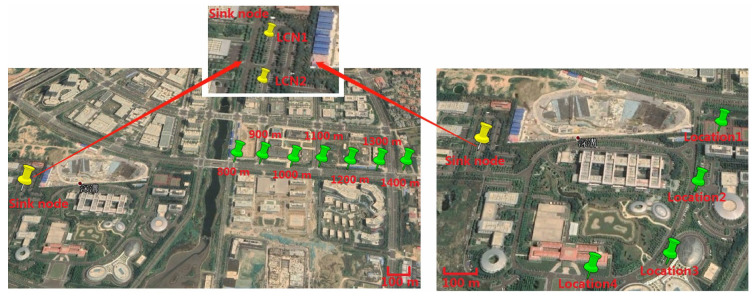
Locations of sensor node and local convergence node in the park scenarios.

**Figure 8:**
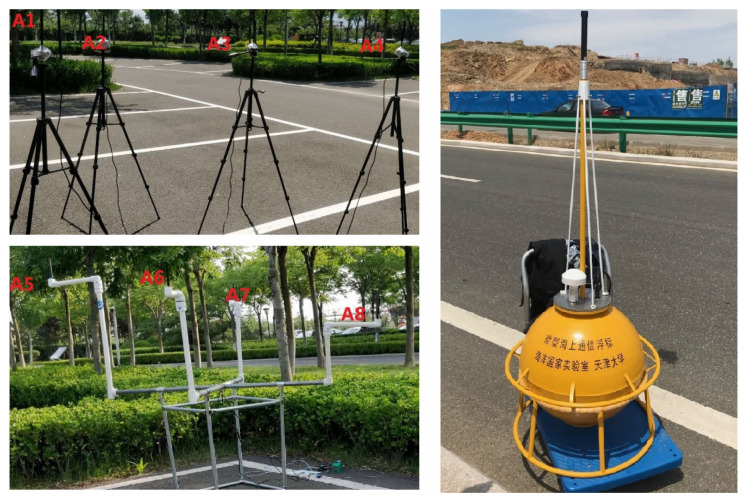
The sink node and the sensor node in the park scenarios.

To analyze the performance of the macrodiversity reception scheme comprehensively, experiments were carried out in the researched park. The two local convergence nodes (LCN1 and LCN2) of the sink node were 30 m apart and placed at an open location in the park. Each local convergence node was equiped with four antennas to form a cluster. The height of antenna was about 1.5 m, and any two antennas were more than one meter apart. Experiments were divided into two parts in the park scenarios. The first experiment analysed performance of macrodiversity reception scheme using different number of antennas and the second experiments compared the performance of diversity scheme using different antenna placement strategies in the park scenarios. We put the sensor node aside by the road for testing during the first measurement. The location of the sensor node was selected 800 m from the sink node and a test location was added every additional 100 m. In the second experiment, we selected four locations in the researched park separately. At each location, we tested the transmission performance of diversity scheme using different antenna placement methods. The specific implementation steps included three parts. First, the two local convergence nodes were about 30 m apart and we validated the performance of macrodiversity reception scheme with two clusters using four antennas in the sink node. Second, the overall eight antennas in the sink node were placed at LCN1 to form a cluster using eight antennas, and the performance of single cluster reception scheme in LCN1 was analyzed. Finally, the overall eight antennas in the sink node were placed at LCN2 to test the performance of the single cluster reception scheme in LCN2. A total of 3000 frames were measured at each location during measurements. The locations of sensor node and local convergence node in the two experiments are shown in Figure 7. The scene of the sink node and the sensor node are shown in Figure 8.

#### 4.2.1. Performance Analysis of Macrodiversity Reception Scheme Using Different Number of Antennas

**Figure 9:**
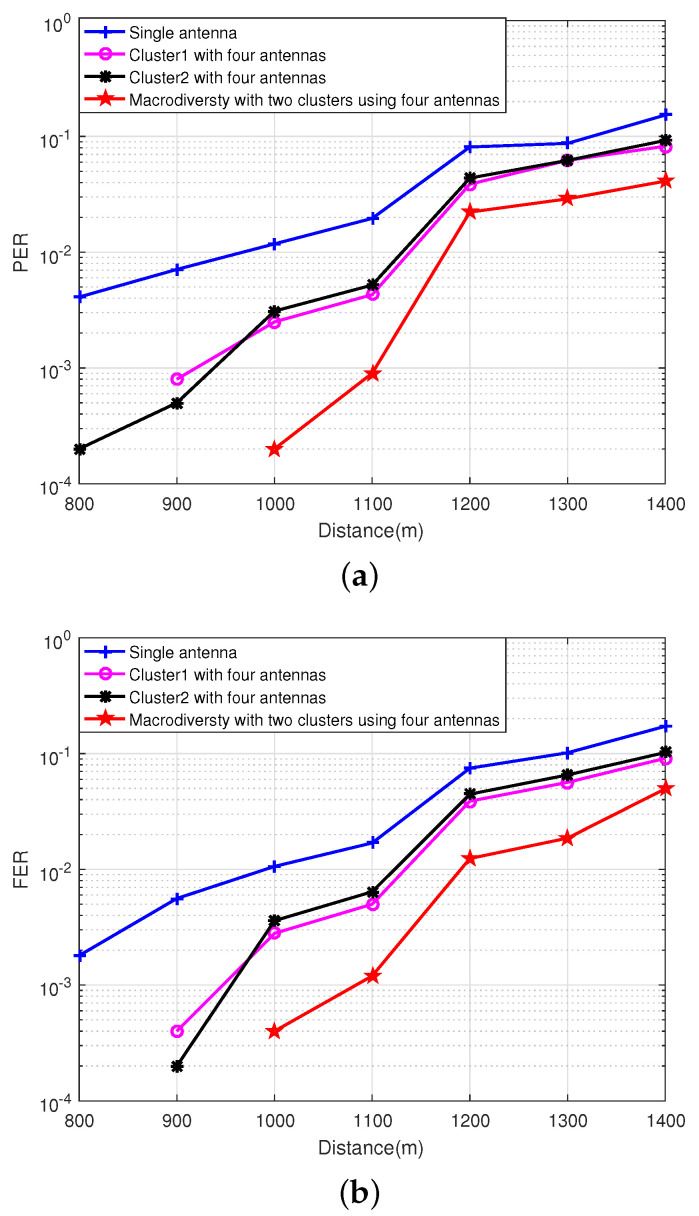
The relationship between performance of macrodiversity reception scheme using different number of antennas and transmission distance. (**a**) The relationship between PER of macrodiversity reception scheme using different number of antennas and transmission distance. (**b**) The relationship between FER of macrodiversity reception scheme using different number of antennas and transmission distance.

The channel fading in the park environment is relatively stable because the sensor node and the sink node are stationary. However, the obstacles between sensor node and sink node are different when the location of the sensor node changes, which causes that the channel fading is different. The first experiment mainly analyzes the relationship between the transmission performance of the macrodiversity reception scheme using different number of antennas and the transmission distance in the park scenarios, and a total of seven locations were tested. All antennas in the sink node are in non-line-of-sight (NLOS) transmission. When the transmission distance is less than 1100 m, there are some trees and small buildings between the sensor node and sink node. When the transmission distance is greater than 1100 m, there are some tall buildings near the sensor node. The experiment analyzes PER and FER of macrodiversity reception scheme with different number of antennas varying with the transmission distance in the park scenarios and the experimental results are plotted in Figure 9.

It can be observed from Figure 9 that the FER of single antenna reception scheme is about $2\times 10^{-3}$ when the transmission distance is 800 m. Both the single cluster reception scheme using four antennas and the macrodiversity reception scheme can achieve error-free transmission. As the transmission distance increases, the performance of the single cluster reception scheme using four antennas scheme begins to decline. When the transmission distance is 1000 m, the FER of the two local convergence nodes are $3\times 10^{-3}$ and $4\times 10^{-3}$ respectively. The macrodiversity reception scheme can still obtain better transmission performance, and its FER is $4\times 10^{-4}$. When the transmission distance is more than 1100 m, the performance of the three reception schemes decrease rapidly, and the gain of macrodiversity reception scheme decreases.

#### 4.2.2. Performance Comparison of Diversity Scheme with Different Antenna Placement Strategies

**Figure 10:**
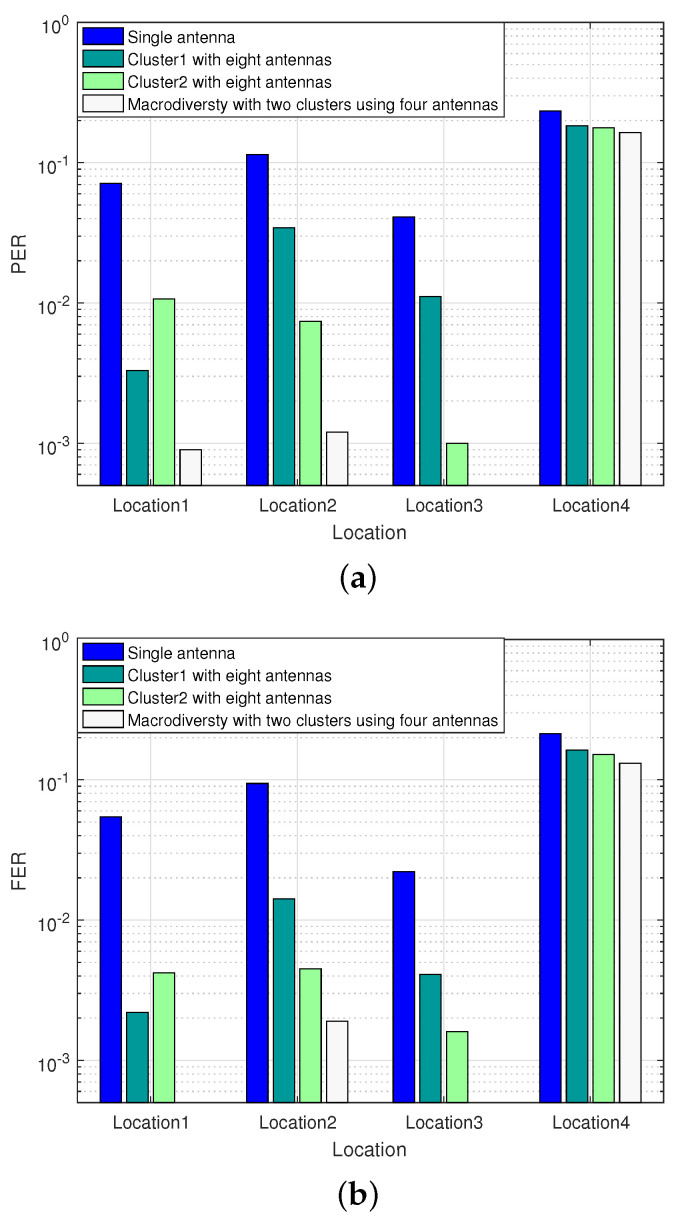
Performance comparison of diversity scheme with different antenna configuration schemes. (**a**) Comparison of PER between between macrodiversity reception scheme with two clusters using four antennas and single cluster using eight antennas reception scheme. (**b**) Comparison of FER between between macrodiversity reception scheme with two clusters using four antennas and single cluster using eight antennas reception scheme.

To compare the transmission gain of single cluster using eight antennas reception scheme with two clusters using four antennas reception scheme, we conducted the second experiment in the researched park. Limited by the size of the park, we reduced the transmitting power of the RF module to 0 dBm. When the sensor node was located in Location1 and Location3, some antennas in the sink node were in LOS transmission. When the sensor node was located in Location2 and Location4, all antennas in the sink node were in NLOS transmission. Especially when sensor node were in Location4, the sensor node was completely obscured by the building. The experiment compares the performance of two clusters reception scheme with single cluster reception scheme using the same number of antennas in the researched park scenarios. The experimental results are shown in Figure 10.

It can be seen from Figure 10 that both two clusters using four antennas reception scheme and single cluster using eight antennas reception scheme can improve transmission performance in the park scenarios. When the sensor node is located in Location1, the transmission between the sensor node and some antennas in sink node are in LOS, and the FER of the single cluster using eight antennas reception scheme placed on the two local convergence nodes is $2\times 10^{-3}$ and $4\times 10^{-3}$ respectively. Under the circumstances, the macrodiversity reception scheme with two antenna clusters can achieve error-free transmission. When the sensor node is located in Location2, there are some obstacles with small occlusion area between the sensor node and sink node, the FER of single cluster using eight antennas reception scheme at LCN1 is $1.5\times 10^{-2}$, while the macrodiversity reception scheme can still guarantee a better transmission performance (FER = $2\times 10^{-3}$). When the sensor node is located in Location4, the sensor node is completely blocked by obstacles, and most of the antennas in the sink node cannot work. Under the circumstance, the performance of the macrodiversity reception scheme is similar to the single cluster using eight antennas reception scheme, and is close to the single antenna reception scheme.

### 4.3. Experiment Results and Analysis in the Ocean Scenarios

The site of experiment in the ocean scenarios was selected at a dock in Qingdao. During the experiment, the buoy equipped with hardware of sensor node was pulled down to a fixed location by the fishing boat and placed in the sea for testing. The sink node was placed on the shore to receive data. Two local convergence nodes of the sink node were 20 m apart. The antennas were fixed by tripods. The height of antenna was about 1.5 m, and any two antennas were more than one meter apart.

**Figure 11:**
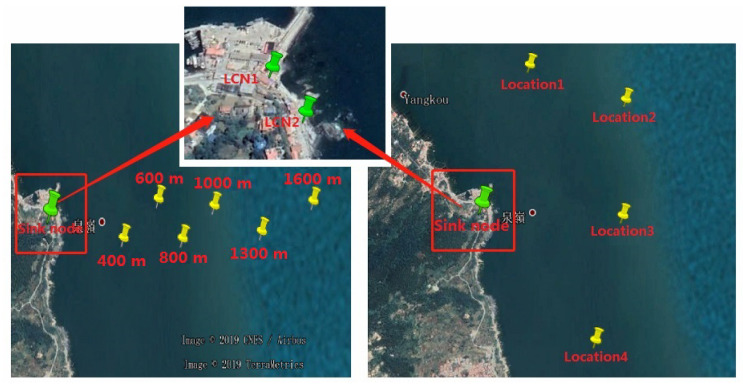
Locations of buoys and sink node in the ocean scenarios.

**Figure 12:**
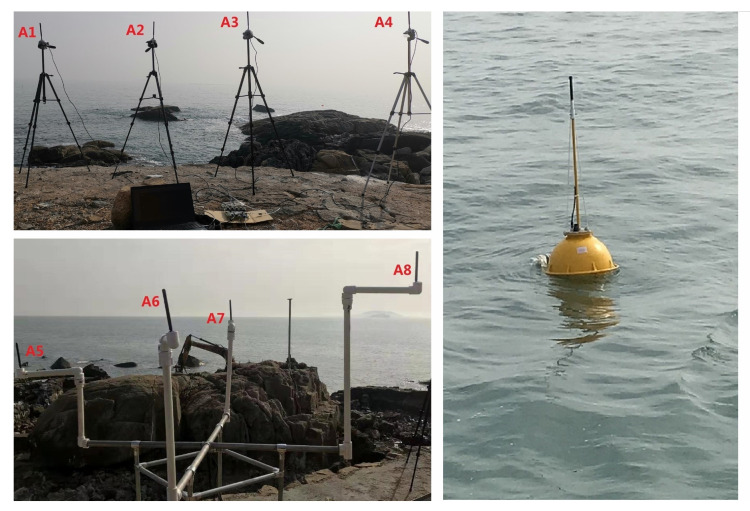
The sink node and the sensor node in the ocean scenarios.

In the third experiment, the sensor node was placed 400, 600, 800, 1000, 1300, and 1600 m offshore for testing. Then, in order to analyze the performance of different antennas placement strategies in the ocean scenarios, we conducted the fourth experiment within a radius of 1100 m. A total of four test locations were selected for the measurement, and the comparison measurement was performed at different locations in the area to be monitored. The experiment tested 3000 frames at each location. The locations of buoys and local convergence nodes in the ocean scenarios are shown in Figure 11. The scene of the sink node and the sensor node in the ocean scenarios are shown in Figure 12. The weather on the day of the experiment was fine, with a southeast wind of levels 3–4.

#### 4.3.1. Performance Analysis of Macrodiversity Reception Scheme Using Different Number of Antennas

**Figure 13:**
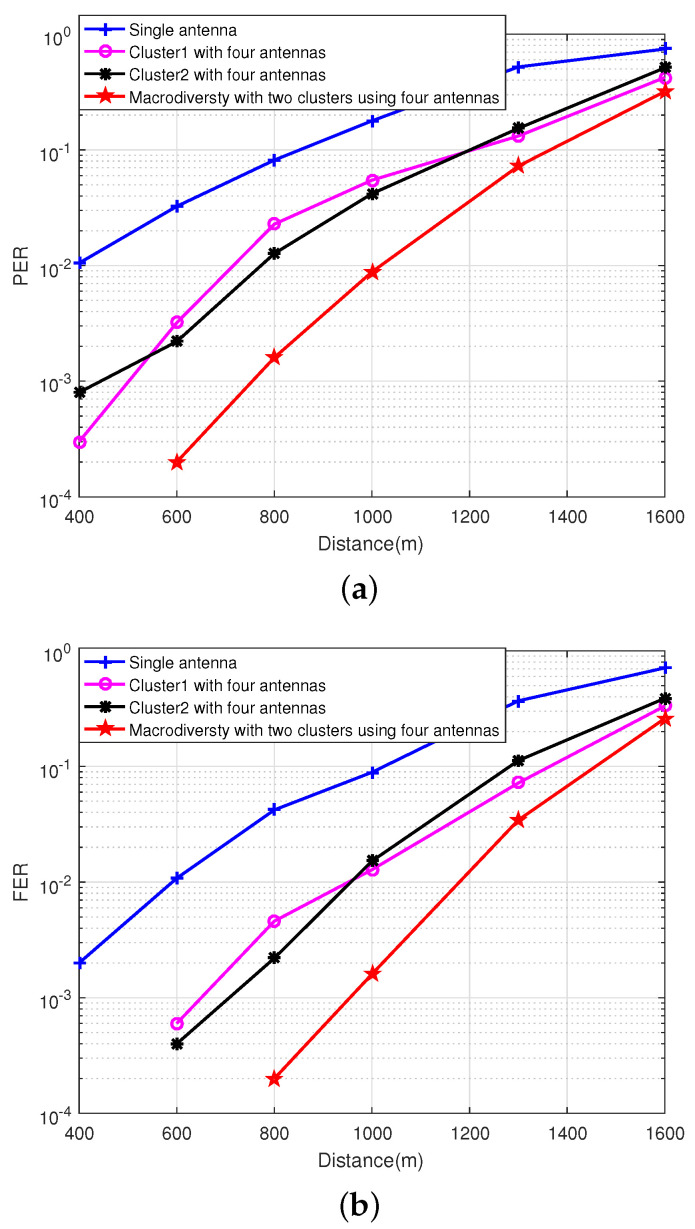
The relationship between performance of macrodiversity reception scheme using different number of antennas and transmission distance. (**a**) The relationship between PER of macrodiversity reception scheme using different number of antennas and transmission distance. (**b**) The relationship between FER of macrodiversity reception scheme using different number of antennas and transmission distance.

Compared with the park environment, the marine environment is relatively open, and there is no blocking object between the sensor node and sink node. However, the reflection plane of the signal changes over time due to the ocean waves. The variation in the height and slope of the ocean waves causes the reflection plane to show different tilt angles, which causes severe multipath effects. The macrodiversity system proposed in this paper configures multiple hard-decision receivers at different locations to receive data. Different paths have different channel fading factors. The combination of multiple receiving copies can greatly improve the success rate of data transmission using diversity. The experiment analyzes the PER and FER of macrodiversity reception scheme with different number of antennas varying with the transmission distance in the ocean scenarios and the experimental results are shown in Figure 13.

As we can see from Figure 13, the macrodiversity reception scheme can obtain 3–4 dB transmission gain compared with single antenna reception scheme at PER = $10^{-2}$. In detail, the macrodiversity reception scheme can achieve error-free reception within 600 m in the ocean scenarios, and still has good reception performance (FER < $10^{-2}$) within 1000 m. With the increase of the transmission distance, the reception performance of each cluster starts to decline, which causes the performance of macrodiversity reception scheme to decrease rapidly.

#### 4.3.2. Performance Comparison of Diversity Scheme with Different Antenna Placement Strategies

**Figure 14:**
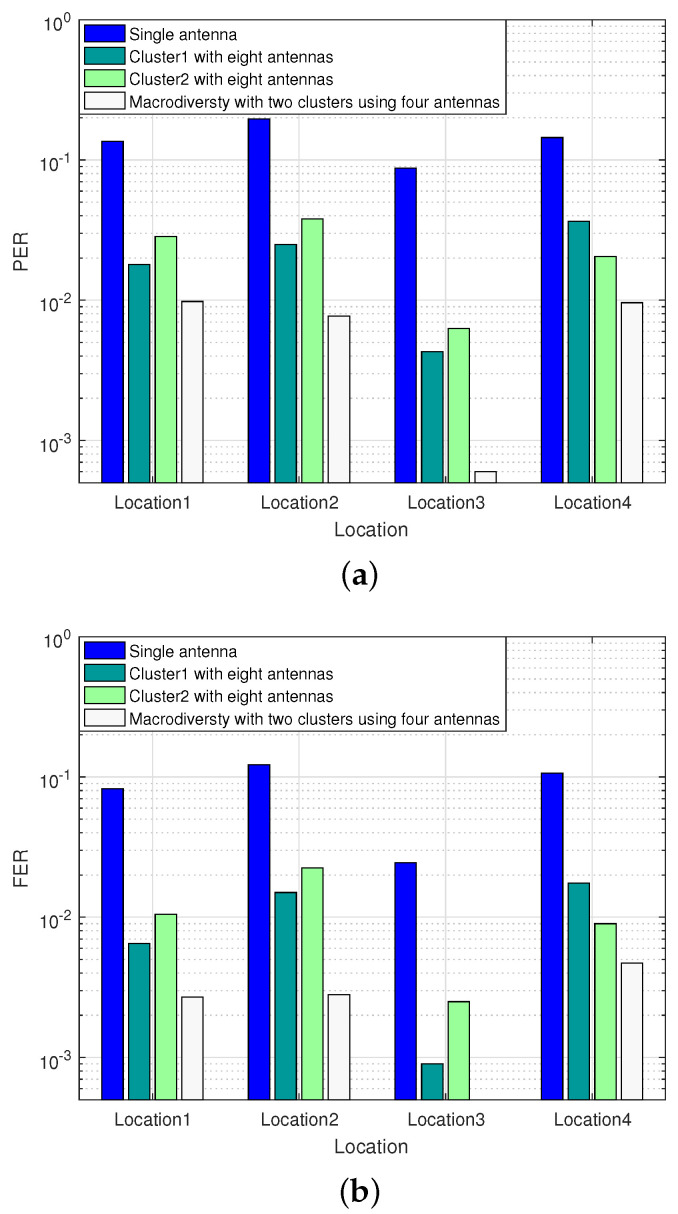
Performance comparison of diversity scheme with different antenna configuration schemes. (**a**) Comparison of PER between macrodiversity reception scheme with two clusters using four antennas and single cluster using eight antennas reception scheme. (**b**) Comparison of FER between macrodiversity reception scheme with two clusters using four antennas and single cluster using eight antennas reception scheme.

The fourth experiment mainly analyzes the PER and FER of diversity scheme with different antenna placement scheme at different locations within a radius of 1100 m. Four locations were selected for testing and the transmission performance of the two reception schemes are analyzed. Among them, the distance from each location to the sink node is as follows: Location1-1000 m, Location2-1100 m, Location3-800 m, Location4-900 m. The experimental results are shown in Figure 14.

It can be seen from Figure 14 that the macrodiversity reception scheme with two clusters can achieve higher reliability transmission (PER < $10^{-2}$) compared with single cluster reception scheme within the transmission distance of 1100 m. When the sensor node is located in Location1 and Location4, the paths from the sensor node to the two local convergence nodes are approximately in a straight line, and the received signals are highly correlated. Under the circumstances, the transmission gain of the macrodiversity reception scheme is close to that of single cluster reception scheme. When the sensor node is located in Location3, the paths from the sensor node to the two local convergence nodes are quite different, and the correlation of signals in the two local convergence nodes is also weak. At this location, the FER of single cluster reception scheme using eight antennas scheme at LCN1 and LCN2 are $9\times 10^{-4}$ and $2.8\times 10^{-3}$ respectively, while the macrodiversity reception scheme can achieve error-free transmission.

## 5. Conclusions

Reliable transmission has become the main challenge of maritime wireless sensor networks due to infinite multipath effects in the ocean scenarios. In this paper, we propose a highly reliable macrodiversity reception scheme based on multiple hard-decision receivers for maritime wireless sensor networks. The scheme indicates that multiple antennas are divided into several clusters placed at different locations in the sink node, and the multiple packages are combined using the cascaded combing scheme in the sink node, which can achieve lower complexity and higher reliability. Furthermore, we construct a maritime wireless sensor networks experiment platform and conduct experiments to verify the performance of the proposed macrodiversity reception scheme in different scenarios. The experimental results show that the proposed macrodiversity reception scheme has a transmission gain of 3–4 dB compared with the single antenna reception scheme at PER = $10^{-2}$ and has higher transmission gain than the single cluster using same number of antennas reception scheme in the ocean scenarios.

## Figures and Tables

**Table 1:** RF module parameters.

Parameters	Value
Radio Module	CC1101
Base Frequency	433 MHz
TX Power	10 dBm
Modulation Mode	GFSK
Preamble Size	8 Bytes
Sync word Size	4 Bytes
Baud Rate	10 kbps
Bandwidth	116 kHz
